# In Vivo Evaluation of the Effect of *Limosilactobacillus fermentum* MC1 and Its EPSs on the Microbiota and Inflammatory Processes in the Mouse Intestine

**DOI:** 10.3390/ijms27052321

**Published:** 2026-03-01

**Authors:** Nina Čuljak, Nada Oršolić, Dyana Odeh, Andreja Leboš Pavunc, Katarina Butorac, Martina Banić, Jasna Novak, Kate Šešelja, Mirela Baus Lončar, Snježana Ramić, Tanja Jurkin, Jagoda Šušković, Blaženka Kos

**Affiliations:** 1 Department of Biochemical Engineering, University of Zagreb Faculty of Food Technology and Biotechnology, Pierottijeva 6, 10000 Zagreb, Croatia; 2 Division of Animal Physiology, Faculty of Science, University of Zagreb, Horvatovac 102a, 10000 Zagreb, Croatia; 3 Department of Molecular Medicine, Ruđer Bošković Institute, Bijenička 54, 10000 Zagreb, Croatia; 4 Department of Oncological Pathology, Clinical Hospital for Tumors, Sestre Milosrdnice University Hospital Centre, Ilica 197, 10000 Zagreb, Croatia; 5 Radiation Chemistry and Dosimetry Laboratory, Ruđer Bošković Institute, Bijenička 54, 10000 Zagreb, Croatia

**Keywords:** *Limosilactobacillus fermentum*, exopolysaccharides, microbiota, anti-inflammatory properties, encapsulation delivery

## Abstract

*Limosilactobacillus fermentum* MC1 is an exopolysaccharide (EPS)-producing strain with previously determined probiotic potential in vitro. This study aimed to investigate the in vivo capacity of the MC1 strain or its EPSs to modulate intestinal microbiota and assess its anti-inflammatory effects in both healthy and dysbiotic conditions. Therefore, *Lb. fermentum* MC1 and its EPSs were administered to a mouse model of dextran sulfate sodium (DSS)-induced colitis (DIC) and to a healthy group, and the effects were observed. Microbiome analysis was used to detect taxonomic differences between treatments. According to the results, administration of the MC1 strain and MC1-EPSs significantly altered gut microbiome composition at different taxonomic levels. The most notable effect was an increased relative abundance of *Firmicutes* and decreased levels of *Candidatus saccharibacteria*. *Llb. fermentum* MC1, and its EPS administration positively affected several disease parameters: reduced disease activity index (DAI), reduced mouse colitis histology index (MCHI), reduced expression of inflammation-related genes and levels of bleeding, and induced polarization of M1 macrophages to the M2-like macrophage phenotype in the DIC mice. These results, along with those related to the induction of antioxidant enzymes and changes in NF-κB-related gene expression, suggest that strain MC1 and MC1-EPSs could be further investigated for their capacity to alleviate DSS-induced histopathological changes and modulate pro-inflammatory cytokine gene expression in colon tissue, which positively correlates with the secretion of inflammatory cytokines, the delay of intestinal inflammation and the maintenance of intestinal barrier function. The obtained data provide a basis for further research into the potential application of intact or microencapsulated *Llb. fermentum* MC1 cells and its EPSs in colitis therapy.

## 1. Introduction

Many health disorders, especially those affecting the gastrointestinal tract (GIT), are accompanied by changes in the composition of the intestinal microbiota. As a result, the development of microbiome-based interventions is increasing, including the use of pre-, pro-, syn- or postbiotics, either as mono- or multistrain preparations, and even the harnessing of a synthetic gut microbiome [1,2,3]. *Lactobacillus* probiotic strains are widely investigated for their ability to modulate the gut microbiota and improve outcomes associated with inflammatory bowel disease (IBD). IBD is a chronic inflammatory disease caused by an immune response to intestinal microbiota and includes Crohn’s disease and ulcerative colitis (UC) [4]. UC is characterized by recurrent inflammation of the mucosa, which can lead to continuous intestinal damage, resulting in an increased risk of colorectal cancer. Currently, there is no known cause for UC, as it has a complex, multifactorial etiopathogenesis involving genetic, environmental and microbial host factors. Since gut microbiome dysbiosis has been shown to play a role in the pathogenesis of UC [5], the potential of well-documented probiotic strains that can beneficially restore the gut microbiome, protect intestinal tissues and regulate the host immune response through the production of various metabolites, such as exopolysaccharides (EPSs), is being investigated [4,6]. In addition, EPSs produced by *Lactobacillus* strains are being studied as potential immunomodulatory effectors in the mechanistic cascade of molecular interactions to alleviate symptoms and induce remission of UC [7].

*Limosilactobacillus fermentum* MC1 was previously isolated from human milk microbiota based on the presence of the typical “ropy” phenotype and specific properties for probiotic selection [8]. These properties included efficient adherence to Caco-2 cells and the ability to inhibit the growth of *Salmonella* species, possibly resulting from the combined activity of bacteriocin and other antimicrobial compounds. Whole-genome sequencing confirmed the presence of putative EPS-encoding genes in the MC1 genome, and functional annotation revealed subsystems associated with EPSs biosynthesis via the Wzx/Wzy-dependent pathway. Structural characterization of the EPSs, achieved by chemical analyses and subsequent 1D and 2D NMR spectroscopy, revealed that the producer strain MC1 synthesizes a mixture of different EPSs containing galactofuranose and glucopyranose residues [9]. An analysis revealed that the MC1-EPSs could play a role in mediating the exclusion of certain pathogens by preventing their adhesion to the Caco-2 cell line [9]. Here, we sought to identify compositional changes in the mice gut microbiota and specific parameters for UC, possibly associated with supplemental *Limosilactobacillus fermentum* MC1 and MC1-EPSs treatment in dextran sulfate sodium (DSS)-induced colitis in C57BL/6J mice. Finally, since probiotic strains encounter numerous obstacles during passage through the digestive system, we explored a strategy to optimize targeted delivery and controlled release of *Llb. fermentum* MC1 cells by evaluating the suitability of micro-alginate and layer-by-layer (LbL) nanoencapsulation methods for MC1 cell entrapment.

## 2. Results and Discussion

### 2.1. Capacity of Llb. fermentum MC1 and MC1-EPSs to Modulate the Intestinal Microbiota

The anti-inflammatory effects of selected EPS-producing *Lactobacillus* strains in gastrointestinal inflammatory conditions have previously been reported. Studies have shown that *Lactobacillus* strains not only alter the expression of inflammatory cytokines in GIT and reduce the severity of inflammation but also strongly impact the intestinal microbiota [2,10,11,12]. Here, the functionality of an EPS-producing *Lactobacillus* strain in alleviating parameters associated with UC, which is characterized by chronic low-grade inflammation, was investigated after administration of EPS-producer MC1 and MC1-EPSs in a mouse model of (DSS)-induced colitis (DIC). C57BL/6 mice are the most common animal model used for the induction of UC with DSS due to the histopathology of UC, which resembles human disease [13]. The group size was selected based on previous studies using a similar animal model, which showed that specific probiotic lactobacilli potently alleviated the symptoms of DIC [14,15]. Therefore, in this study, interventions with *Lactobacillus* and MC1-EPSs were designed as a feasibility study, minimizing the number of mice following the 3R principle. Also, the present study targeted only the V3 hypervariable region of the 16S rRNA gene. Although this region is widely used for bacterial community profiling, sequencing of a single hypervariable region limits taxonomic resolution, particularly at the species level, which consequently affects taxonomic assignments. First, to confirm a murine model of gut microbial dysbiosis, we treated the mice with DSS and performed compositional analysis of the gut microbiota before and after colitis induction. A significant decrease was observed in the relative abundance of the genera *Barnesiella*, *Allobaculum*, *Bacteroides*, *Prevotella*, *Turicibacter* and *Parasutterella* (Figure S1). In contrast, the relative abundance of the genera *Staphylococcus*, *Shigella* and *Clostridium* significantly increased. Together, these results confirm that DSS treatment significantly affected the relative abundances of several bacterial groups within the intestinal microbiota, inducing marked compositional changes indicative of UC-associated dysbiosis [16,17,18,19,20,21]. DSS rapidly and easily destroys the intestinal barrier, causes mucosal erosion, stimulates the inflammatory response, and, as confirmed here, alters the intestinal microbiome.

Compared to the control group, the *Llb. fermentum* MC1-treated DIC group showed a statistically significant decrease (Figure S2a,b) in the relative abundance of the phyla *Candidatus saccharibacteria* (*p* < 0.0001) and *Chlamydiae* (*p* < 0.01). Conversely, there was a statistically significant increase in the relative abundance of the phyla *Bacteroidetes* on the 3rd day of feeding (*p* < 0.05), *Actinobacteria* on the 5th day after the end of feeding (*p* < 0.05) and *Firmicutes* on the 6th day of feeding. Interestingly, relatively high levels of *Llb. fermentum* were observed even on day 5 after the end of treatment (*p* < 0.001). These findings support the probiotic potential of *Llb. fermentum* MC1, as its administration in DIC mice was associated with an increase in the relative abundance of bacterial phyla involved in maintaining homeostasis and a decrease in the relative abundance of phyla typically abundant in an imbalanced gut microbiota. At the phylum level, a similar trend was observed after MC1-EPS supplementation in the DIC mice group (Figure S2c). A statistically significant increase in the relative abundance of the *Bacteroidetes* phylum was observed after day 6 of feeding (*p* < 0.05), *Firmicutes* at all time points (*p* < 0.001 for day 3, *p* < 0.05 for day 6 of feeding and *p* < 0.001 for day 5 after the end of EPS feeding) and *Actinobacteria* on day 5 after the end of feeding (*p* < 0.0001). In contrast, *Candidatus saccharibacteria* and *Chlamydiae* (*p* < 0.001 and *p* < 0.002, respectively) and *Proteobacteria* on the 6th day after the start (*p* < 0.01) and 5th day after the end (*p* < 0.01) of feeding decreased markedly. These changes in the composition of the intestinal microbiota may possibly result from the prebiotic action of EPSs.

**Figure 1:**
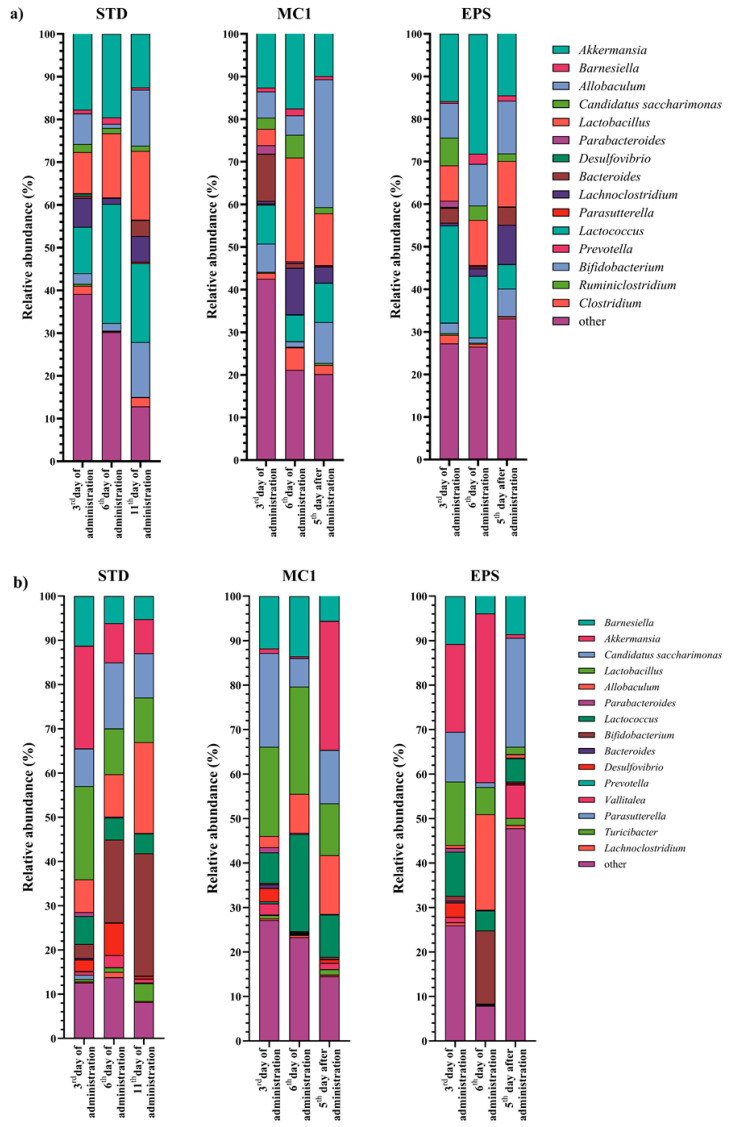
Relative abundance (%) of bacteria at the genus level in feces of (**a**) mice with dextran sulfate sodium (DSS)-induced colitis (DIC) and (**b**) healthy mice fed with a standard laboratory diet (STD), strain *Llb. fermentum* MC1 (MC1) or EPSs of *Llb. fermentum* MC1 (EPS). Samples were taken on the 3rd, 6th, and 11th day of feeding for the control group and on the 3rd and 6th day of feeding and the 5th day after termination of feeding for the MC1 and EPS groups.

A total of nine genera were significantly affected by *Llb. fermentum* MC1 supplementation in the DIC experimental model (Figure 1a). This was evident by a global increase in the relative abundance of *Allobaculum*, *Lactobacillus*, *Bacteroides*, *Lactococcus* and *Turicibacter*, while the proportion of *Desulfovibrio*, *Chlamydia*, *Candidatus saccharimonas* and *Staphylococcus*, detected at least at one time point, decreased. EPS administration in DIC mice resulted in a significant increase in the relative abundance of *Allobaculum*, *Bifidobacterium*, *Alistipes* and *Lactococcus* and a sporadic decrease in the relative abundance of *Desulfovibrio*, *Candidatus saccharimonas*, *Staphylococcus* and *Chlamydia* (Figure 1a).

A comparison was also made of the results from in vivo tests on the effect of feeding with *Llb. fermentum* MC1 or its EPSs on the composition of the gut microbiota of healthy mice compared to mice fed the STD (control sample). The phyla *Bacteroidetes* and *Firmicutes* make up the majority of the gut microbiota of healthy mice (Figure S3a), where a decrease in the relative abundance of the phylum *Bacteroidetes* was observed in healthy mice fed the STD during the different time points (*p* < 0.01 for the 3rd, *p* < 0.001 for the 6th and *p* < 0.001 for the 11th day of feeding). When healthy mice were fed *Llb. fermentum* MC1 (Figure S3b), a significant increase in the relative abundance of the phyla *Firmicutes* was observed at different time points (*p* < 0.05 for the 3rd day of feeding, *p* < 0.001 for the 6th day of feeding and *p* < 0.05 for the 5th day after the end of feeding) and of *Verrucomicrobia* on the 5th day after the end of *Lactobacillus* administration (*p* < 0.05). Oppositely, a decrease in the relative abundance of the phylum *Candidatus saccharibacteria* was observed after the 6th day of feeding (*p* < 0.01). When healthy mice were fed with EPSs produced by *Llb. fermentum* MC1 (Figure S3c), a significant increase in the relative abundance of *Firmicutes* (*p* < 0.05 for the 6th day of feeding and *p* < 0.001 for the 5th day after the end of feeding), *Verrucomicrobia* (*p* < 0.01) and *Actinobacteria* (*p* < 0.01 for the 6th day of feeding and *p* < 0.05 for the 5th day after the end of feeding) was observed. A decrease in the relative abundance of *Candidatus saccharibacteria* was observed after the 6th day of treatment (*p* < 0.0001).

When healthy mice were fed the STD (Figure 1b), a decrease in the relative abundance of the genera *Barnesiella*, *Prevotella* and *Parasutterella* was observed. When healthy mice were fed the *Llb. fermentum* MC1 strain (Figure 1b), the relative abundance of the genera *Akkermansia*, *Lactobacillus*, *Lactococcus* and *Eubacterium* significantly increased at least at one time point compared to the control. After the healthy group was fed MC1-EPSs (Figure 1b), the relative abundance of the genera *Akkermansia*, *Allobaculum, Bifidobacterium*, *Ruminococcus* and *Eubacterium* increased, while the relative abundance of the genus *Bacteroides* decreased at least at one time point compared to before feeding.

**Figure 2:**
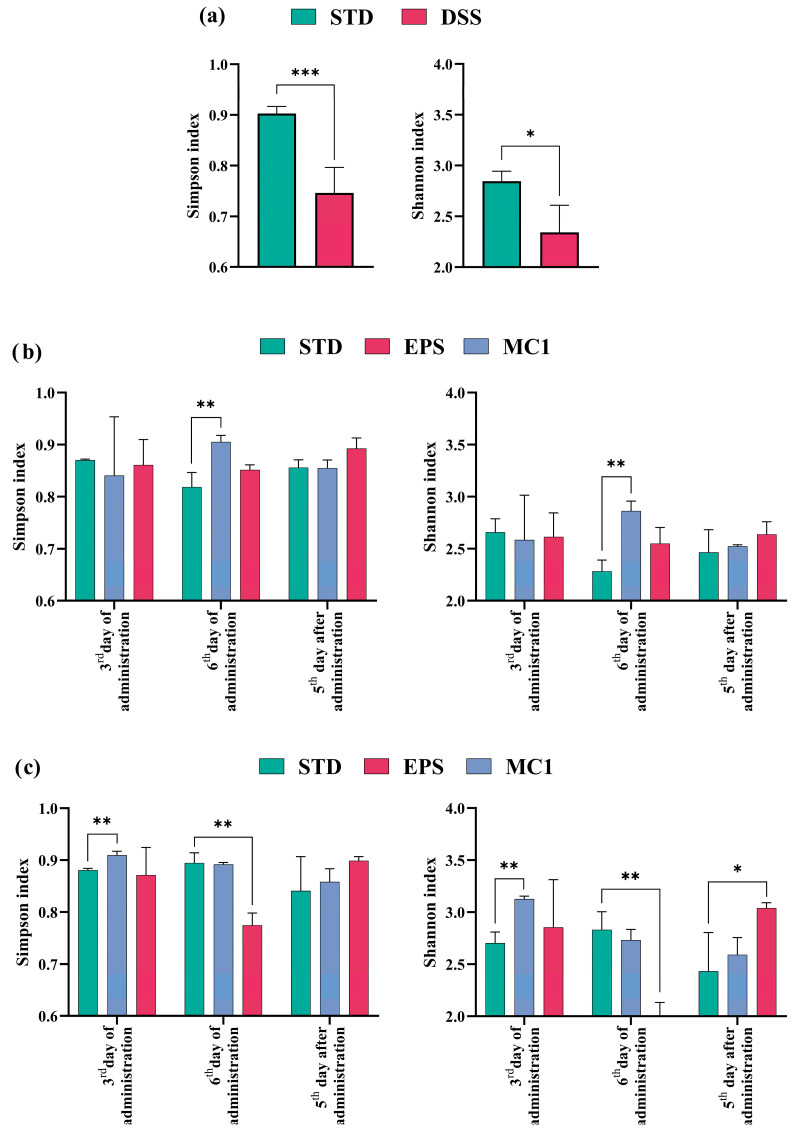
α-Diversity (**a**) before (STD) or after (DSS) colitis induction; (**b**) in diseased and (**c**) healthy mice fed with standard laboratory diet (STD) and *Limosilactobacillus fermentum* MC1 (MC1) or its EPSs at different time points, expressed by “Simpson” (**left**) and “Shannon” (**right**) metrics. Statistically different compared to STD group (* *p* < 0.05, ** *p* < 0.01, *** *p* < 0.001).

To assess the impact of *Llb. fermentum* MC1 or extracted MC1-EPSs on the intestinal microbiota, PAST software was used for diversity analysis by calculating the Simpson and Shannon indices (Figure 2). The results suggest that microbiota composition changed significantly after DSS treatment, as a significant decrease in α-diversity expressed by the Simpson (*p* < 0.001) and Shannon (*p* < 0.05) indices was observed (Figure 2a), reflecting a loss of species richness in the microbiome of mice with DIC. Reduced microbial diversity due to intestinal dysbiosis is associated with a weakened immune system and increased incidence of disease [2,22,23]. α-Diversity of the intestinal microbiota in DIC mice was significantly higher in the *Llb. fermentum* MC1-fed group on the 6th day compared to the control group, as determined by the Simpson (*p* < 0.01) and Shannon (*p* < 0.01) diversity indices (Figure 2b). As for the healthy mice (Figure 2c), *Llb. fermentum* MC1 supplementation on the 3rd day resulted in a statistically significant increase in the Simpson and Shannon indices (*p* < 0.01) compared to the control. The Simpson and Shannon indices (*p* < 0.01) were also determined after the addition of EPS. According to the obtained results, on the 6th day of treatment, lower Simpson’s and Shannon’s indices (*p* < 0.01) were found compared to mice fed the STD, while on 5th day after administration an increase was observed (*p* < 0.05).

Together, these results indicate that DSS treatment altered microbiota composition by reducing α-diversity, while *Llb. fermentum* MC1 and its EPSs were associated with partial restoration of microbial diversity, suggesting their potential modulatory effect on the gut microbiome.

**Figure 3:**
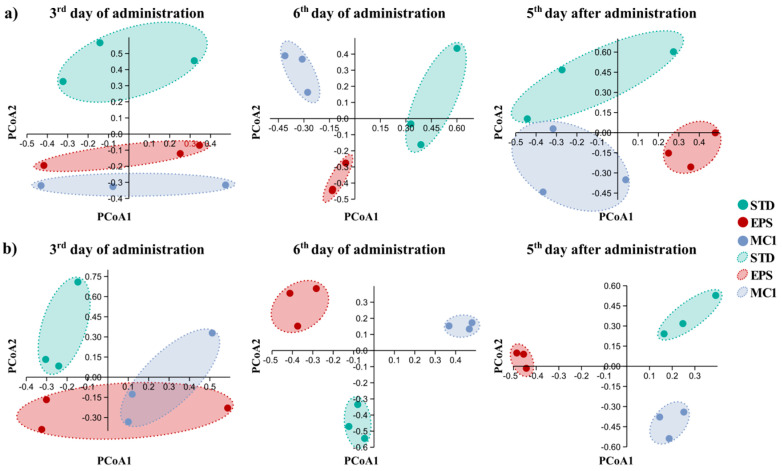
Beta diversity analysis of gut microbiota over a time in (**a**) DIC and (**b**) healthy mice as a Principal Coordinate Analysis (PCoA) plot based on Bray–Curtis dissimilarity.

Further, Principal Coordinate Analysis (PCoA) based on Bray–Curtis dissimilarity was used to visualize beta-diversity of the gut microbiota in the DIC and healthy group (Figure 3). In both DIC and healthy mice, samples clustered primarily according to treatment group within each experimental period, indicating that treatment had an effect on microbial community composition. Notably, in healthy mice, samples in the last two time periods clustered more tightly by treatment than those of diseased mice in the same periods, suggesting a more consistent or pronounced treatment-associated shift in microbial community structure in healthy mice compared to diseased mice.

To be noted, because technical replicates were used in this study, biological variability between individual samples could not be fully assessed. Therefore, the findings primarily reflect overall community trends rather than inter-individual variability. Future studies using full-length 16S sequencing or shotgun metagenomics, combined with biological replication, would provide improved taxonomic resolution and functional insight into microbial community dynamics.

### 2.2. Llb. fermentum MC1 and MC1-EPSs Attenuate DSS-Induced Histopathological Changes and Gene Expression of Pro-Inflammatory Cytokines in the Colon Tissue

Gut microbial metabolites and cell structures, and thus inanimate as well as intact cells, play key roles in inflammatory signaling, interacting both directly and indirectly with host immune cells. Since metagenomic analysis confirmed the capacity of *Llb. fermentum* MC1 and MC1-EPSs to modulate microbiota composition, we also monitored their ability to alter the inflammatory response and promote the regeneration of the intestinal mucosa in DIC mice. After 5-day administration of DSS, the development of colitis was evident from clinical signs resembling those of the human form of the disease. It is hypothesized that the negatively charged sulfate groups damage the intestinal mucosa and epithelial cells, leading to an increase in gut permeability. Disruption of intestinal permeability by DSS allows microorganisms and their derivatives to invade through the loosened epithelial tight junctions (TJs) and activate intestinal macrophages, which consequently secrete inflammatory cytokines and chemokines that recruit immune cells such as dendritic cells, T and B cells and neutrophils that stimulate colon inflammation processes [24].

Intestinal damage caused by DSS and the accumulation of inflammatory cells, lymphocytes and granulocytes in the submucosal and mucosal layers are visible in histopathological preparations of colon tissue stained with HE (Figure S4). DSS induced edema, mucosal erosions, and disrupted epithelial layers with focal severe dysplasia were also observed; even carcinoma developed in two animals. The mouse colitis histology index (MCHI) score for the DSS group was 16.50 ± 0.50 (range 13–18), while administration of EPS and especially MC1 in the DIC group reduced the histology scores by 11.33 ± 1.30 and 5.67 ± 1.23, respectively (Table 1). Application of EPSs, and especially MC1 in the DIC group, reduced the severity of colitis as determined by MCHI by 31.33% (*p* > 0.05) and 65.64% (*p* < 0.001), respectively. Administration of MC1 and EPSs resulted in significantly fewer lesions in the mucosal area, with reduced infiltration of inflammatory cells and decreased ulcerative sores. However, localized severe dysplasia and signs of colitis, including crypt shortening, inflammation and microabscesses, were still observed in animals treated with EPSs but were less pronounced in animals treated with MC1. These changes suggest that MC1 and EPSs attenuate DSS-induced colonic damage by improving epithelial integrity.

**Figure 4:**
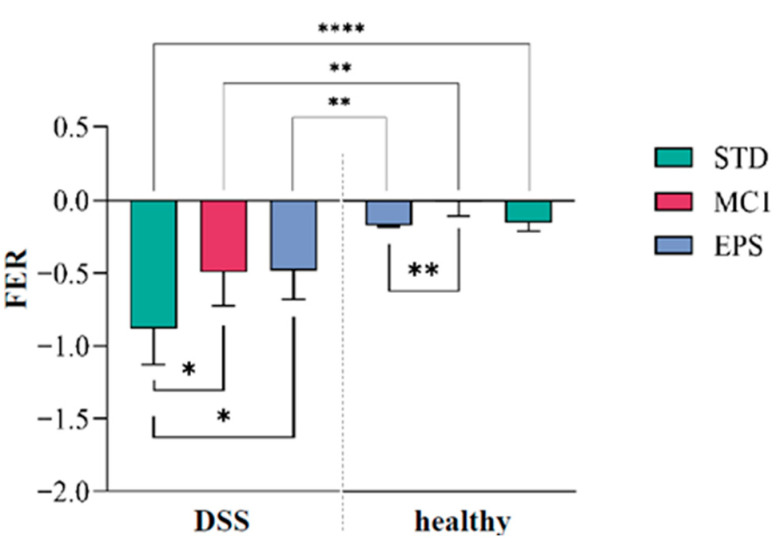
Food efficiency ratio (FER) in mice with DSS-induced colitis and healthy mice fed with the standard laboratory diet (STD) with or without the addition of *Limosilactobacillus fermentum* MC1 (MC1) or its EPSs (MC1-EPS). Statistically different (* *p* < 0.05, ** *p* < 0.01, **** *p* < 0.0001).

Food intake and weight loss were also monitored to assess whether the treatments affected changes in appetite and FER (Figure 4). In the group of DIC mice fed STD (DSS + STD), a significantly lower (*p* < 0.05) FER was observed in comparison to DIC mice fed EPSs (DSS + EPS) or *Llb. fermentum* MC1 (DSS + MC1), while higher FER in comparison to mice fed STD was observed only in healthy mice fed *Llb. fermentum* MC1 (*p* < 0.01). DSS treatment affected the progression of the inflammatory process, and a decrease in body weight was observed across all animal groups (Table S1), indicating mucosal damage and reduced nutrient uptake. In addition, a statistically significant decrease in body weight and FER was observed in all tested groups of DIC mice compared to the groups of healthy mice. Overall, MC1 and EPSs improved nutrient utilization and mitigated DSS-induced weight loss. Min et al. [25] showed that the application of EPSs isolated from the *Lactiplantibacillus plantarum* YW11 strain resulted in less weight loss in the treated groups than in the untreated groups. Our results are consistent with these findings, as at the end of the experiment, the DIC group of mice showed a significantly lower FER than the groups treated with *Llb. fermentum* MC1 and MC1-EPSs. As expected, food utilization was improved in healthy mice compared to mice with DIC.

**Figure 5:**
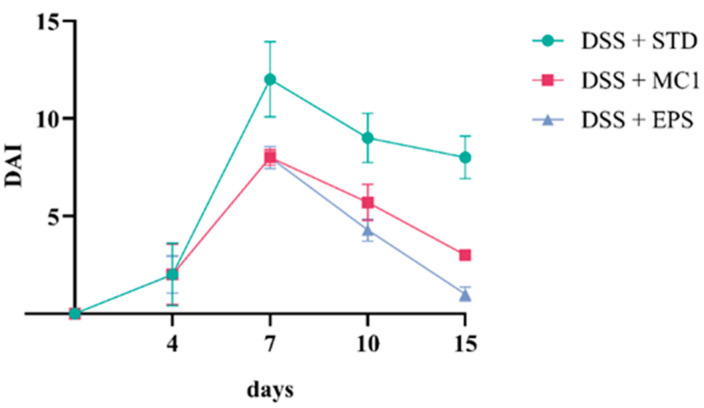
Disease activity index (DAI) in mice with dextran sulfate sodium (DSS)-induced colitis (DIC) fed a standard laboratory diet (STD) before and after supplementation with *Llb. fermentum* MC1 (MC1) or MC1-EPSs.

In addition to the FER, the DAI was also determined using the monitored parameters of weight, fecal consistency and presence of blood in the feces. Weight loss, presence of blood in the feces and changes in fecal consistency were observed. As expected, the DAI value increased as inflammation progressed and the clinical condition worsened. Treatment with *Llb. fermentum* MC1 positively affected disease progression by slowing it and improving recovery after colitis induction; however, treatment with its EPSs had an even better effect, as evidenced by the lowest DAI value (Figure 5). Butorac et al. [2] also confirmed a reduction in the DAI in DIC mice after supplementation of *Lpb. plantarum* strain D13 and demonstrated a reduction in the inflammatory process compared to the control group.

One of the most common extraintestinal systemic complications of IBD is anemia [26]. Anemia is closely associated with immune system dysregulation, leading to increased cytokine levels and blood loss from the inflamed colon. Furthermore, reduced absorption of iron (Fe) and nutrients and the incidence of anorexia are present in severe UC due to increased intestinal motility. To investigate the effects of *Llb. fermentum* MC1 or EPSs on maintaining mucosal integrity, Fe absorption, and preventing the development of anemia as a result of UC, hematological blood analysis was performed (Table 2). In the group of DIC mice treated with EPSs (DSS + EPS), there was a statistically significant increase in the number of erythrocytes (*p* < 0.01), hemoglobin concentration (*p* < 0.01) and hematocrit (*p* < 0.05) in the blood compared to the DIC group (DSS + STD). According to the data obtained, EPSs can reduce anemia in UC, suggesting that EPSs have increased antioxidant capacity, reduce mucosal damage and consequently increase Fe absorption from enterocytes. In the group of DIC mice treated with the MC1 strain (DSS + MC1), a statistically significant decrease was found only in mean corpuscular hemoglobin (MCH) (*p* < 0.05) compared to the DIC group, indicating insufficient Fe absorption. Considering that all other hematopoietic parameters are slightly elevated in the MC1-treated group compared to the DSS group, the reduction in MCH alone does not indicate a serious anemia disorder. However, it should be noted that treatment with EPSs has a higher hematopoietic value compared to MC1, especially a significantly higher hemoglobin value (*p* < 0.01). It is clear that mice treated with MC1 or EPSs alleviate the consequences of DSS, improve the absorption of Fe and nutrients by maintaining intestinal integrity and reduce bleeding. As for the healthy mice, a statistically significant increase in thrombocyte concentration (*p* < 0.01) was observed in the EPS-treated group compared to the mice fed the STD, but still within the reference interval (155.55–1036.88 × 10^9^/L) (Table S2).

To determine the severity of the inflammation in the different groups of mice, the differential blood count was analyzed, including the percentage of leukocytes–granulocytes (neutrophils, eosinophils, and basophils) and agranulocytes (lymphocytes and monocytes) (Table 3). The results show that the proportion of neutrophils was significantly reduced in the groups of DIC mice treated with the strain MC1 (DSS + MC1) or EPSs (DSS + EPS) compared to the DSS control group (*p* < 0.01). This finding is consistent with the DAI, as the significantly increased neutrophil count in the DSS group indicates disease progression (Figure 4). On the contrary, a significant increase in the proportion of monocytes (*p* < 0.01) was observed in the DSS + MC1 group, suggesting a potential antitumor effect of the MC1 strain, as it has been shown that the increased monocytes in the blood and the decreased lymphocytes may result from an inflammatory response against tumor growth [27]. Another possible explanation for the increased accumulation of macrophages in the MC1-treated DIC group may be their role in the regeneration process and in establishing tissue homeostasis after DSS-induced tissue damage. The inflammatory process and the accumulation of macrophages in damaged tissue are crucial for removing necrotic cells and faster tissue regeneration after severe DSS-induced damage. Macrophages, through phagocytosis and the digestion of damaged parts of tissue, cells and microorganisms, for which they have powerful lysosomal enzymes, prepare the tissue for ending the inflammatory process. In addition to cell clearance, these inflammatory cells stimulate the division of healthy cells around the necrotic area, which is crucial for successful tissue regeneration. In the MC1-treated group, a decrease in the proportion of lymphocytes (*p* < 0.05) and an increase in the proportion of monocytes (*p* < 0.05) were observed compared to the group treated with MC1-EPSs alone. The lower monocyte-to-lymphocyte ratio in the EPS-fed groups compared to MC1 suggests a potential benefit of consuming EPSs, as a higher ratio is associated with inflammation or even tumors. In healthy mice treated with MC1 or EPSs, a difference in eosinophil count was observed, with higher counts in mice fed the MC1 strain than in mice fed EPSs (Table S3). Our data are consistent with those of Al-Okbi et al. [28], who showed that anemia and Fe deficiency with elevated oxidative stress in the colon are associated with inflammatory biomarkers and an impaired immune system in DIC compared to EPS- or MC1-fed mice or normal healthy mice.

To determine the degree of inflammation, RNA was isolated from colon tissue, followed by a gene expression analysis of inflammatory markers (IL-1β, IL-6, MCP-1, IL-1α, TLR4, TNF-α, CD68, and TGF-β), molecular markers of apoptosis (BCL2, AIFM1, and IGF1R), ER (CHOP and GRP94) and an oxidative stress (NOX2) marker (Figure S5). The results show a statistically significant increase in the relative expression of IL-6 (*p* < 0.01), MCP-1 (*p* < 0.05), IL-1α (*p* < 0.05), TNF-α (*p* < 0.05), CD68 (*p* < 0.01), TGF-β (*p* < 0.05), GRP94 (*p* < 0.01) and NOX2 (*p* < 0.05) genes and a decrease in CHOP expression (*p* < 0.0001) in DIC mice. An increase in the expression of pro-inflammatory cytokines indicates inflammation, which is expected since acute colitis, considered a form of IBD, was induced. In addition to pro-inflammatory cytokines, the concentration of the chemokine MCP-1, which is secreted by macrophages and attracts a large number of monocytes to the site of inflammation, also increases during the inflammatory process [29].

**Figure 6:**
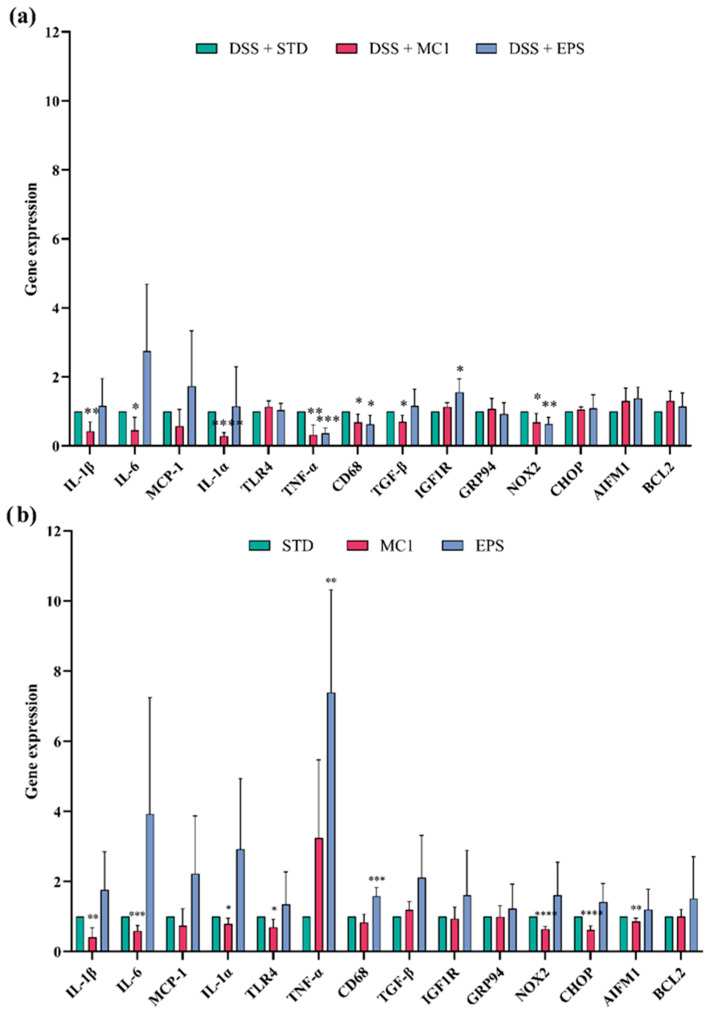
Expression of genes related to inflammation (IL-1β, IL-6, MCP-1, IL-1α, TLR4, TNF-α, CD68, and TGF-β), apoptosis (BCL2, AIFM1, and IGF1R), and endoplasmic (CHOP, GRP94) and oxidative stress (NOX2) in the colon of (**a**) DSS-treated mice on standard laboratory diet (STD), fed with strain *Limosilactobacillus fermentum* MC1 or its EPSs, and (**b**) healthy mice fed with MC1 or its EPSs. Expression of monitored genes was compared relative to expression in standard-diet-fed colitis-affected mice (expression level set to 1). Statistically different compared to the STD group (* *p* < 0.05, ** *p* < 0.01, *** *p* < 0.001, **** *p* < 0.0001).

To determine whether the severity of inflammation changes between the group of DIC mice and the group of DIC mice subsequently treated with *Llb. fermentum* MC1 or its EPSs, the gene expression of several markers related to inflammation, apoptosis, and ER and oxidative stress was monitored. Treatment of DIC mice (Figure 6a) with MC1 resulted in a statistically significant decrease in the expression of IL-1β (*p* < 0.01), IL-6 (*p* < 0.05), IL-1α (*p* < 0.001), TNF-α (*p* < 0.01), CD68 (*p* < 0.05) and TGF-β (*p* < 0.05), as well as inflammatory M1 macrophage marker genes and oxidative stress molecular marker NOX2 (*p* < 0.05). Treatment with EPSs significantly reduced the expression of inflammatory markers TNF-α (*p* < 0.001) and CD68 (*p* < 0.05) and oxidative stress molecular marker NOX2 (*p* < 0.01) and increased the expression of molecular marker IGF1R (*p* < 0.05), indicating an anti-apoptotic effect and increased cell survival. EPSs were isolated from the fraction of the cell-free bacterial culture supernatant and purified by NaOH precipitation, followed by extensive dialysis, which contains soluble EPSs assumed to not contain structural components of the bacterial cell wall. Altogether, these results support the potential antioxidant activity of the potential probiotic strain MC1 and MC1-EPSs, as a statistically significant decrease in the expression of NOX2 was observed. The findings that EPSs reduce the production of certain inflammatory cytokines are also consistent with findings that EPSs can reduce anemia in UC. Based on the data obtained, administration of MC1 and EPSs could be associated with the alleviation of certain symptoms of DIC, accompanied by increased antioxidant activity, polarizing macrophages from M1 to M2, and changes in nuclear factor kappa B (NF-κB)-related gene expression. These alterations may be linked to the modulation of inflammatory cytokine secretion and intestinal inflammation in mice, potentially contributing to the maintenance of TJ integrity [30,31,32]. Differences in immune cell response to cytokine production between mice treated with MC1 or MC1-EPSs may be partly due to differences in Fe absorption in DSS-damaged mucosa and intracellular Fe levels in immune cells, including macrophages, due to an Fe-dependent mechanism in modulating the TLR signaling pathway [33,34]. Min et al. [25] showed that EPSs isolated from *Lpb. plantarum* strain YW11 reduced inflammation in DIC mice by enhancing the expression of IL-10 and decreasing the levels of pro-inflammatory cytokines TNF-α, IL-1β, IL-6, IL-12, IL-18, and INF-γ. Similar results were obtained by Liu et al. [35], who showed that EPSs of *Lb. helveticus* reduce inflammation by regulating cytokine levels, improving barrier integrity and modulating the intestinal microbiota. Yang et al. [36] also demonstrated that *L. ruminis* FXJWS27L3 can alleviate DIC, likely related to the reduction of pro-inflammatory cytokine expression, upregulation of short-chain fatty acids, and restoration of gut microbiota balance. Based on the data obtained, the reduced level of inflammatory cytokines and the strengthening of the regenerative capacity of the mucosa suggest that treatment with *Llb. fermentum* MC1 or its EPSs plays a role in protecting the mucosa, maintaining the integrity of the intestinal barrier by reducing the inflammation of damaged surrounding cells, stimulating mucosal regeneration and reducing the bleeding process, as indicated by higher levels of hematopoietic determinants and a slower index of disease development. According to our data, MC1 and EPSs can also successfully alleviate DIC by reducing the ratio of M1/M2 macrophages in the liver, kidney and spleen, as indicated by reduced NO levels, an important indicator of macrophage polarization. Thus, MC1 and EPSs act as mediators in switching the M1 polarization of macrophages toward the M2 phenotype (Figure 6).

**Figure 7:**
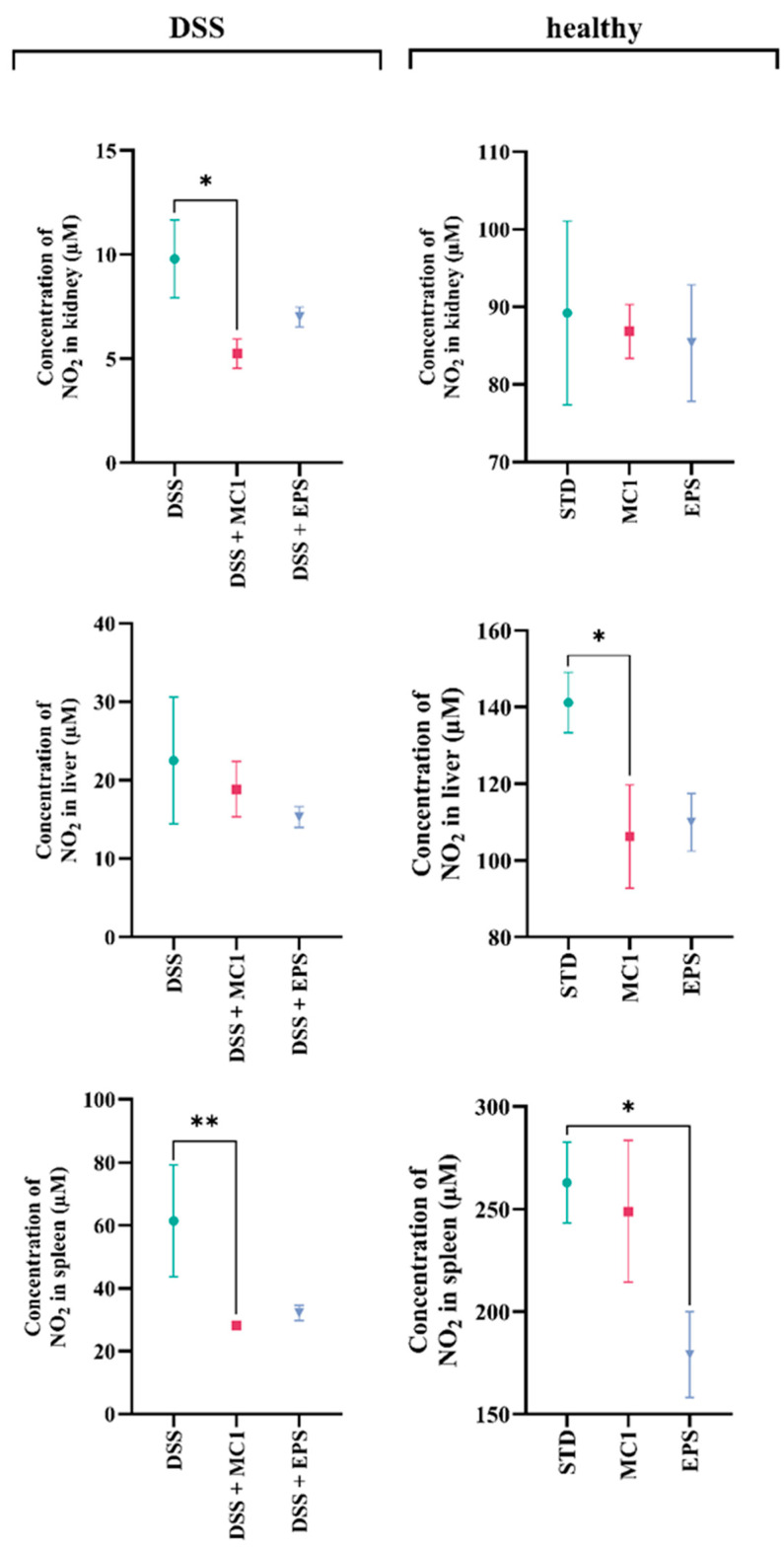
Nitric oxide (NO) concentration (μM) in the kidney, liver and spleen of DSS-treated and healthy mice on the standard laboratory diet (STD) fed with strain *Limosilactobacillus fermentum* MC1 or MC1-EPSs. Statistically different compared to the DSS or STD group (* *p* < 0.05, ** *p* < 0.01).

Treatment of healthy mice (Figure 6b) with MC1 alleviated inflammation, as evidenced by a statistically significant decrease in the expression of inflammatory markers IL-1β (*p* < 0.01), IL-6 (*p* < 0.001), IL-1α (*p* < 0.05), TLR4 (*p* < 0.05), CD68 (*p* < 0.05) and TGF-β (*p* < 0.05), as well as molecular markers associated with oxidative stress, NOX2 (*p* < 0.0001), ER stress CHOP (*p* < 0.0001) and AIFM1 apoptosis (*p* < 0.01). Furthermore, the MC1 strain is associated with a significant inhibition of AIFM1 expression by regulating CHOP and GADD34 expression, which act as major regulators of ER-stress-induced apoptosis [37,38]. However, EPS treatment significantly increased the expression of the inflammatory marker CD68 (*p* < 0.001), indicating the activation of macrophages in response to various inflammatory stimuli, including the LPS toxin. EPSs show a strong immunomodulatory effect, manifested by increased expression of TNF-α, which stimulates cytotoxic lymphocytes and cytokine secretion, thereby increasing the body’s resistance to infections. In summary, MC1 treatment appears to modulate inflammation by promoting the polarization of macrophages toward the M2 phenotype, which is confirmed by the NO concentration (Figure 7).

In summary, the in vivo data show that EPSs and MC1 effectively alleviate DIC symptoms, including weight loss, colonic symptoms related to stool formation and fecal bleeding, DAI, anemia, and inflammatory colon pathology. These effects were accompanied by changes in the expression of inflammation-related cytokines and NF-κB-associated genes, suggesting potential involvement of these pathways in immune regulation and intestinal barrier integrity. Reduced release of M1 cytokines after treatment of DIC mice with MC1 and EPSs is essential for intestinal barrier repair. In healthy mice, EPSs led to non-specific activation of the host immune system, while MC1 helps maintain colon tissue homeostasis. The anti-inflammatory effects of *Lbl. fermentum* MC1 and its EPSs are probably mediated through a combination of direct immune modulation and secondary alterations in gut microbial composition. However, the relatively small number of animals per group underscores the need for future studies with larger cohorts to further confirm the findings and assess the validity of further preclinical investigations.

### 2.3. Enhancing the Viability of Llb. fermentum MC1 by Encapsulation Methods

We have demonstrated that consumption of live *Llb. fermentum* MC1 bacteria can respond to microenvironmental challenges in vivo and result in beneficial effects in the DIC model, as evident from MC1 strain–host interactions. However, the strain’s activity during large-scale production can be compromised during manufacturing and storage and ultimately when applied in the host in situ under unfavorable conditions in the digestive tract. Gastric fluid, digestive enzymes and bile salts in the small intestine are critical factors that reduce probiotic cell numbers, thereby limiting colonization and proliferation in the GIT and subsequently impairing their function [39,40]. To overcome these limitations, different systems for delivery are being investigated, with encapsulation techniques being the most prominent [41].

Accordingly, the following encapsulation experiments were designed as a preliminary methodological approach to improve the stability, survival, and delivery efficiency of the *Llb. fermentum* MC1 strain under stressful conditions and also for possible testing of encapsulated probiotic cells for subsequent in vivo studies. We tested two approaches: the extrusion technique of microencapsulation of MC1 cells in alginate, both with and without the addition of galactooligosaccharides (GOS) or fructooligosaccharides as supportive prebiotic substrates, and the LbL method for nanoencapsulation of MC1 cells. A high percentage of microencapsulation of *Llb. fermentum* MC1 was achieved, with no statistical difference among the matrices applied, whether with alginate alone or with FOS or GOS The success of nanoencapsulation of *Llb. fermentum* MC1 was monitored by measuring the zeta potential, a parameter that refers to the electrokinetic potential in colloidal systems and affects the stability and behavior of encapsulated products in the digestive tract [42]. Zeta potential values (mV) evidenced a successful nanoencapsulation of *Llb. fermentum* MC1 using the LbL method, as a trend of random exchange of negative and positive charge was observed after individual measurements (Figure S6).

**Figure 8:**
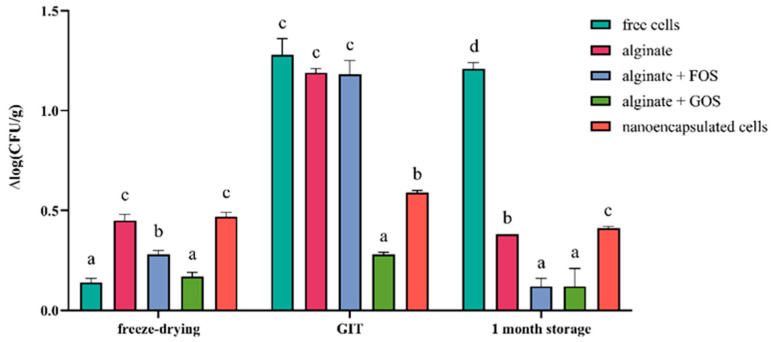
Cell mortality [Δlog(CFU/g)] of free, microencapsulated in alginate, with or without addition of FOS (fructooligosaccharides) or GOS (galactooligosaccharides) and nanoencapsulated *Limosilactobacillus fermentum* MC1 cells after freeze-drying, exposure to simulated gastrointestinal tract (GIT) conditions and 1-month storage. Different letters indicate statistical difference between data across exposure to different conditions (*p* < 0.05).

The survival of free, encapsulated and nanoencapsulated *Llb. fermentum* MC1 cells after freeze-drying, exposure to simulated GIT conditions and one month of storage was investigated (Figure 8). Non-encapsulated (free) cells and alginate-encapsulated cells with the addition of GOS survived freeze-drying with approximately equal cell counts; however, other treatments were also effective in protecting against cell number reduction. As for survival in simulated GIT conditions, the results show that microencapsulation in alginate with added GOS most effectively protected *Llb. fermentum* MC1 cells, as cell mortality [Δlog(CFU/g)] was the lowest. During one month of storage, the lowest mortality was observed after microencapsulation of *Llb. fermentum* MC1 in alginate with the addition of GOS and FOS. These findings correlate with the results of Liao et al. [43], who demonstrated that *Llb. fermentum* L7 also exhibits better survival rates under simulated GIT conditions and during long-term storage after microencapsulation in alginate, especially with the addition of various prebiotics.

## 3. Materials and Methods

### 3.1. Conditions for Maintaining and Growing Lactobacillus Strain

EPS-producing *Llb. fermentum* MC1 was originally isolated from human milk microbiota [8]. The whole-genome sequence of *Llb. fermentum* MC1 is deposited in the NCBI database under the accession number SAMN22155537 (BioProject PRJNA388578). The strain was isolated and is part of the Culture Collection of the Laboratory for Antibiotic, Enzyme, Probiotic and Starter Culture Technologies, University of Zagreb, Faculty of Food Technology and Biotechnology. MC1 strain stock was maintained at −80 °C in MRS broth (BD Difco, Detroit, MI, USA) with 15% (*v*/*v*) glycerol (Sigma-Aldrich, St. Louis, MO, USA). Prior to the experiment, strain MC1 was subcultured twice under microaerophilic conditions in MRS broth at 37 °C overnight.

### 3.2. Experimental Animals and Study Design

**Figure 9:**
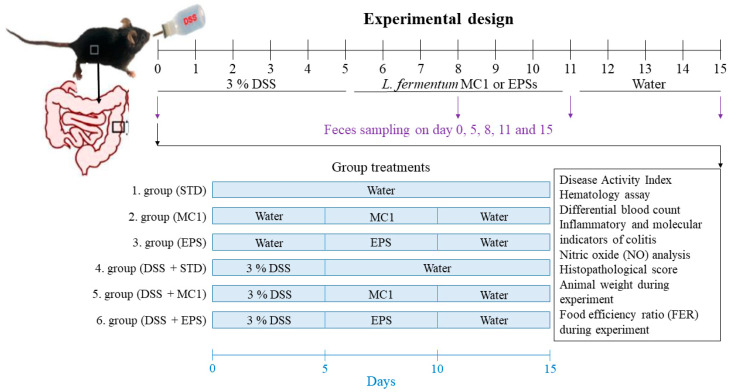
Schematic representation of the experimental groups of mice used in the study, including healthy controls and DSS-induced colitis (DIC) mice, along with their corresponding diets and treatments: 1. group (STD)—healthy mice fed with standard laboratory diet (STD); 2. group (MC1)—healthy mice fed with STD and *Llb. fermentum* MC1; 3. group (EPS)—healthy mice fed with STD and MC1-EPSs; 4. group (DSS + STD)—DSS-treated mice fed with STD; 5. group (DSS + MC1)—DSS-treated mice fed with STD and *Llb. fermentum* MC1; 6. group (DSS + EPS)—DSS-treated mice fed with STD and MC1-EPSs.

The 2–3-month-old male C57BL/6 mice, which were initially individually marked, weighing 20–30 g upon admission, were divided into six groups of 6 animals with approximately equal body weight (±3 g) and kept under controlled conditions. Groups 1, 2 and 3 of healthy mice were fed a standard laboratory diet (STD); group 2 additionally received *Llb. fermentum* MC1, while group 3 received MC1-EPSs. Groups 4, 5 and 6 of C57BL/6 mice were first treated with 3% DSS (36,000–50,000 Da; MP Biomedicals, Santa Ana, CA, USA) in sterile water to induce acute colitis over 5 days [2]. In accordance with the treatments of healthy groups, the DIC groups were fed in the same manner: STD (group 4), STD supplemented with *Llb. fermentum* MC1 (group 5) and STD supplemented with MC1-EPSs (group 6) (Figure 9). Groups fed with *Llb. fermentum* MC1 received 0.5 mL of a cell suspension containing 1 × 10^9^ CFU/dose/day, and those fed with MC1-EPSs received 0.5 mL of EPSs at a concentration of 1 mg/mL via intragastric cannula for 5 days. The concentration of EPSs used for in vivo analysis was selected based on our previous study, where this concentration significantly reduced the adhesion of *Escherichia coli* 3014 to the Caco-2 cell line in vitro [8]. The EPS extraction included recovery from the bacterial culture supernatant followed by a purification step achieved by NaOH precipitation and later dialysis. The fraction obtained contains soluble EPSs and is expected to be free of insoluble bacterial cell wall components [8]. General mouse health status, which included checking movement, alertness, coat texture and the presence of discharge from the nose, eyes, mouth, and ears, was observed regularly. Five days after the completion of treatments, mice were injected intraperitoneally with an anesthetic mixture containing 75 mg/kg Narketan^®^ (Vetoquinol S.A., Lure, France; active substance Ketamine) and 10 mg/kg Xylapan^®^ (Vetoquinol Biowet Sp., Gorzów Wielkopolski, Poland; active substance Xylazine) for sampling required in further analysis.

The research was approved by the Committee of Bioethics and Animal Welfare of the University of Zagreb, Faculty of Science (No. 251-58-10617-19-285; date of approval: 12 April 2019), the Croatian National Ethics Committee for the Protection of Animals Used for Scientific Purposes and the Ministry of Agriculture of the Republic of Croatia (No. 525-10/0255-19-5, date of approval: 8 August 2019). The research was conducted in accordance with the ethical principles of the Republic of Croatia, in accordance with the Regulation on the Protection of Animals Used for Scientific Purposes (Official Gazette No. 55/2013; Animal Protection Act and Official Gazette No. 102/17), and by following the Guide for the Care and Use of Laboratory Animals, DHHS Publ. (NIH) # 86-23, 2011.

### 3.3. Fecal Microbiota Compositional Analysis

Fecal samples were collected with sterile forceps from the housing cages before treatment with DSS, after treatment/before administration, on days 3 and 6 of administration and 5 days after administration of *Llb. fermentum* MC1 or EPS. The samples were stored at −80 °C until use. DNA from the mice fecal samples was isolated and analyzed according to Butorac et al. [2]. Briefly, total metagenomic DNA was extracted using a Maxwell^®^ DNA Tissue Kit (Promega, Madison, WI, USA) according to the manufacturer’s instructions. DNA was quantified using a BioSpec-Nano spectrophotometer (Shimadzu, Kyoto, Japan) and stored at −20 °C until use. 16S rRNA sequencing was conducted using an Illumina MiSeq platform by amplifying primers 341F (5′-CCTACGGGNGGCWGCAG-3′) and 518R (5′-ATTACCGCGGCTGCTGG-3′) at Molecular Research LP (MRDNA, Shallowater, TX, USA). Sequencing on the Illumina MiSeq platform yielded 10,765,620 paired-end reads. All raw 16S rRNA sequencing data generated in this study have been deposited in the NCBI Sequence Read Archive (SRA) under BioProject accession number PRJNA1424430 at https://www.ncbi.nlm.nih.gov/sra/PRJNA1424430 (accessed on 13 February 2026). Beta-diversity analysis was performed using Bray–Curtis dissimilarity matrices, followed by Principal Coordinate Analysis (PCoA) for ordination and visualization of community differences. All alpha- and beta-diversity analyses were conducted using PAST software (version 4.03).

### 3.4. Physiological Parameters in C57BL/6J Mice

#### 3.4.1. Body Weight

Body weight was measured using a digital scale (Electronic balance ABS 220-4, Kern&Sohn, Balingen-Frommern, Germany). The percentage of weight loss was calculated for the animals as follows:

Weight change (%) = [(Final weight − Initial weight)/Final weight] × 100

Additionally, the food efficiency ratio (FER) was determined as follows:

FER = (Weight gain/Daily food intake)

#### 3.4.2. Disease Activity Index Assessment

The disease activity index (DAI) score, based on daily observation of the frequency and intensity of inflammatory markers, was used to assess DSS-induced colitis. The following parameters were determined: (a) weight loss (0 = none, 1 = 1–3% weight loss, 2 = 3–6% weight loss, 3 = 6–9% weight loss and 4 ≥ 9% weight loss); (b) stool consistency/diarrhea (0 = normal, 2 = loose stool, 4 = watery diarrhea); and (c) bleeding (0 = no bleeding, 2 = mild bleeding, 4 = heavy bleeding). DAI values, calculated as the total sum of the above parameters, can range from 0 (unchanged) to 12 (severe colitis).

#### 3.4.3. Histologic Injury Score

To assess histological alterations in the distal colon, tissue samples were fixed in 4% paraformaldehyde, embedded in paraffin, and cut into 5 μm thick sections. The slides were then stained with hematoxylin and eosin (HE), analyzed by a pathologist using an Axiostar plus microscope (Carl Zeiss Microscopy GmbH, Jena, Germany) at a magnification of 200× and photographed with a Zeiss Axiocam 105 color camera (software Zeiss Lite 2.3; Zeiss, Gottingen, Germany).

The pathohistological assessment according to Koelink et al. [44] includes the following parameters: the presence of ulcerations, inflammatory cells (such as neutrophils, macrophages, lymphocytes, and plasma cells), signs of edema, loss of crypts, hyperplasia of surface epithelial cells, reduction of goblet cells and signs of epithelial regeneration. The extent of ulcerative lesions was assessed using the mouse colitis histology index (MCHI), which includes eight histological components: inflammatory infiltrate, goblet cell loss, hyperplasia, crypt density, muscle thickness, submucosal infiltration, ulcerations, and crypt abscesses (all categorized from 0 to 3). The quantitative assessment by MCHI was performed systematically on all samples, and the scoring results reflect the overall range and severity of lesions within each group. The histopathologic score was calculated as the sum of the eight scores, ranging from 0 to 24, and the MHCI was determined by two blinded observers.

MCHI = 1 × Goblet Cell Loss (Four Categories) + 2 × Crypt Density (Three Categories) + 2 × Hyperplasia (Four Categories) + 3 × Submucosal Infiltrate (Four Categories))

#### 3.4.4. Detection of Nitric Oxide (NO) via the Griess Reaction

The NO concentration was measured using a Griess reagent system kit (Promega, Madison, WI, USA) according to the method described by Odeh et al. [45]. The Griess method is based on a diazotization reaction in an acidic environment of phosphoric acid, where NO_2_- reacts with sulfanilamide and creates a diazonium cation to which N-1-naphthylethylenediamine dihydrochloride binds, resulting in a red-colored azobenzene product that is measured spectrophotometrically at 540 nm using an Infinite^®^ 200 PRO spectrophotometer (Tecan, Männedorf, Switzerland). Nitric (II) oxide concentration was measured on homogenized samples of liver, kidney and spleen. An amount of 0.1 M sodium nitrite dissolved in water, with concentrations ranging from 0 to 100 μM, was used as a standard. From the standard curve of absorbance versus nitrite concentration, the slope line was determined, and the concentration of NO_2_ in the samples, expressed as μM/μL, was calculated via the slope line.

### 3.5. Hematological Assay

Blood samples were collected from the heart according to the guidelines of the Clinical Laboratory Standards Institute [46] and the World Health Organization [47]. For hematological analyses, blood samples were collected in ethylenediaminetetraacetic acid (EDTA) vacutainers (Abaxis, Union City, CA, USA). Estimation of the number of erythrocytes, leukocytes and thrombocytes, hematocrit, hemoglobin, volume of erythrocytes and thrombocytes, concentration and content of hemoglobin in erythrocytes and distribution of erythrocytes was determined using the Horiba ABX169 automated analyzer (Micros, Issy-les-Moulineaux, France).

### 3.6. RNA Isolation, cDNA Synthesis and qPCR

RNA was isolated from murine colon tissue using a NucleoSpin^®^RNA kit (Macherey-Nagel, Düren, Germany), and cDNA was synthesized using a High-Capacity cDNA Reverse Transcription Kit (Applied Biosystems, Darmstadt, Germany), following the manufacturer’s instructions. The obtained cDNAs were used in a quantitative polymerase chain reaction (qPCR) according to a method based on SYBR Green (Thermo Fischer Scientific, Waltham, MA, USA) dye as described in Šešelja et al. [48]. Two reference genes, β-actin (ACTβ) and β2-microglobulin (B2M), were used for normalization. The expression of the following genes was analyzed: GRP94—glucose-regulated protein 94; IGFR1—insulin-like growth factor 1 receptor; IL1α—interleukin 1α; IL1β—interleukin 1β; IL6—interleukin 6; MCP1—monocyte chemoattractant protein 1; NOX2—NADPH oxidase 2; TLR4—Toll-like receptor 4 receptor; TNFα—tumor necrosis factor α; TGFβ—transforming growth factor β; CD68 antigen; CHOP—protein homologous to CCAAT-enhancer-binding protein; BCL2—B-cell lymphoma protein 2; and AIFM1—apoptosis inducing factor mitochondria associated 1. Specific primer pairs and qPCR conditions are provided in Table S4.

The amplification results of target genes by qPCR were analyzed using the REST^©^ (Relative Expression Software Tool) MCS^©^ software v2 with the ΔΔCt method. Relative gene expression (R) was calculated using the following equation:

$$R=\frac{{{(E}_{target\;gene})}^{∆Ct\;target\;gene(A\;control\;-\;A\;sample)}}{{{(E}_{ref.gene})}^{∆Ct\;ref.gene(A\;control\;-\;A\;sample)}}$$

### 3.7. Micro- and Nanoencapsulation of Llb. fermentum MC1

Microencapsulation of *Llb. fermentum* MC1 was performed according to the procedure previously described by Čuljak et al. [49]. In summary, *Llb. fermentum* MC1 cells were encapsulated in alginate, with or without the addition of prebiotic substrates GOS or FOS, via the extrusion technique.

Nanoencapsulation of *Llb. fermentum* MC1 was performed using the LbL method according to Anselmo et al. [50] with modifications. After overnight growth and washing of *Llb. fermentum* MC1 cells, the cells were resuspended in a positively charged polyelectrolyte poly (diallyl dimethylammonium chloride) (PDDA; 2 mg/mL; Sigma-Aldrich, USA) solution and incubated for 10 min. After incubation, the suspension was centrifuged at 4200 rpm for 5 min and the cell pellet was washed twice with sterile distilled water. The washed cells were then resuspended in a negatively charged polyelectrolyte poly (sodium styrenesulfonate) (PSS; 2 mg/mL; Sigma-Aldrich, USA) solution and incubated for 10 min. After incubation, the suspension was centrifuged at 4200 rpm for 5 min and the sediment of bacterial cells was washed twice with deionized water to obtain cells with a single polyelectrolyte layer. To obtain nanocapsules with three layers, this process was repeated two more times. To confirm the formation of layers, zeta potential was measured using a Zetasizer Ultra (Malvern Panalytical, Malvern, UK) equipped with a 632.8 nm He-Ne laser, using electrophoretic light scattering in DTS1070 folded capillary cells. For this purpose, 20 µL of the sample was mixed with 2 mL of distilled water and dispersed by vortexing, and 1 mL of this suspension was transferred to a cell. The values of zeta potential are given as the mean value of three measurements.

Micro- and nanocapsules of *Llb. fermentum* MC1 were suspended in 10% (m/V) skim milk (Fluka, Seelze, Germany), freeze-dried in an Alpha 1-2 LD plus freeze dryer (Martin Christ, Osterode am Harz, Germany) and examined for their viability after freeze-drying, exposure to simulated GIT conditions and 1-month storage at +4 °C according to Čuljak et al. [49]. Before determining the number of colony-forming units (CFUs), microencapsulated cells were released from the capsules with 2% Na-citrate (Kemika, Zagreb, Croatia). Non-encapsulated (free) cells served as the control.

### 3.8. Statistical Analysis

For microbiota analysis, fecal samples from all animals within each treatment group were collected and pooled, since the mice were housed together in the same cage and it was not possible to assign fecal pellets to individual animals. Three independent subsamples from each pooled sample were analyzed, representing technical replicates. For other measured parameters (hematology, histopathology, NO concentrations, and gene expression), biological replicates were used, with each measurement performed on individual animals. The results are presented as the mean ± standard deviation. Statistically significant differences among samples were determined by one-way analysis of variance (one-way ANOVA) using Tukey’s honestly significant difference test for post hoc analysis of variance pairwise comparisons (https://www.statskingdom.com/index.html, accessed on 1 July 2024). Immunological parameters were analyzed using the non-parametric Kruskal–Wallis test. Differences between samples were considered significant if *p* < 0.05.

## 4. Conclusions

This study demonstrates that the administration of *Llb. fermentum* MC1 and MC1-EPSs profoundly modified microbiota composition and is associated with their influence on the inflammatory response and regeneration of the intestinal mucosa in DIC mice. Moreover, *Llb. fermentum* MC1 and its EPSs possess anti-inflammatory and antioxidant properties, which contribute to alleviating inflammatory activity and subsequently inducing the healing effect of the gut barrier in DIC mice. Their potential to protect the integrity and regeneration of the intestinal mucosa was confirmed by the reduction in the DAI, MCHI, inflammation-related gene expression, and the bleeding process and by polarizing M1 macrophages to an M2-like phenotype in the mice with DIC. Certainly, the underlying molecular mechanisms of these interactions have to be fully elucidated. Lastly, by demonstrating that microencapsulation of cells in alginate with the addition of GOS is the most efficient approach to deliver a high number of viable *Llb. fermentum* MC1 cells, we pave the way to test the efficacy of microencapsulated MC1 cells in the DIC model. Overall, although limited by being a small-scale animal study and a sequencing approach targeting a single V3 region, these findings encourage further research into the effects of *Llb. fermentum* MC1 and MC1-EPSs on molecular inflammatory, apoptosis and endoplasmic reticulum stress markers, as well as oxidative stress, which could further define the therapeutic potential of this probiotic strain and its EPSs in UC.

## Figures and Tables

**Table 1:** Mouse colitis histology index (MCHI) in DSS-induced colitis (DIC) mice fed with the standard laboratory diet (STD), with or without the addition of *Limosilactobacillus fermentum* MC1 (MC1) or MC1 exopolysaccharides (EPS).

Parameters	DSS + STD	DSS + MC1	DSS + EPS
Goblet cell loss	1.83 ± 0.17 (1–2)	0.67 ± 0.21 (0–1) *	0.67 ± 0.21 (0–1) *
Crypt density	2.00 ± 0.00 (2–2)	0.33 ± 0.21 (0–1) **	1.50 ± 0.22 (1–2)
Crypt hyperplasia	2.33 ± 0.21 (2–3)	0.67 ± 0.21 (0–1) **	1.33 ± 0.21 (1–2)
Muscle thickening	2.17 ± 0.17(2–3)	0.67 ± 0.21(0–1) **	1.17 ± 0.17(1–2)
Submucosal infiltrate	2.00 ± 0.00 (2–2)	1.00 ± 0.26 (0–2) *	1.67 ± 0.21 (1–2)
Score	16.50 ± 0.50 (15–18)	5.67 ± 1.23 (0–8) ***	11.33 ± 1.31 (7–15)

Statistically different compared to DSS + STD group (* *p* < 0.05, ** *p* < 0.01, *** *p* < 0.001). DSS + STD: DIC mice fed standard laboratory diet (STD); DSS + MC1: DIC mice fed STD and *Llb. fermentum* MC1; DSS + EPS: DIC mice fed STD and EPSs of *Llb. fermentum* MC1.

**Table 2:** Hematological parameters in dextran sulfate sodium (DSS)-induced colitis (DIC) mice fed with standard laboratory diet (STD) before and after supplementation with *Llb. fermentum* MC1 (MC1) or MC1-EPSs.

Hematological Parameters	DSS + STD	DSS + MC1	DSS + EPS
Erythrocytes (10^12^/L)	6.35 ± 0.17	6.71 ± 0.26	7.81 ± 0.18 **
Leukocytes (10^9^/L)	3.73 ± 0.37	3.27 ± 0.32 ^ΔΔ^	5.13 ± 0.27
Hemoglobin (g/L)	96.25 ± 2.17	96.83 ± 3.69 ^Δ^	117.83 ± 2.95 *
Hematocrit (%)	0.35 ± 0.02	0.40 ± 0.01	0.46 ± 0.01 **
Erythrocyte volume (fL)	55.07 ± 3.18	59.37 ± 1.19	59.07 ± 1.04
Mean corpuscular hemoglobin (pg)	15.18 ± 0.17	14.40 ± 0.00 *	15.10 ± 0.06
Mean corpuscular hemoglobin concentration (g/L)	279.83 ± 15.07	243.33 ± 4.81	255.67 ± 5.67
Erythrocyte distribution (%)	22.83 ± 0.66	20.63 ± 2.65	21.60 ± 1.16
Thrombocytes (10^9^/L)	431.08 ± 32.41	431.67 ± 14.36	534.33 ± 38.45
Thrombocyte volume (fL)	6.92 ± 0.13	6.63 ± 0.09	6.73 ± 0.06

Statistically different compared to DSS + STD group (* *p* < 0.05, ** *p* < 0.01). Statistically different compared to DSS + EPS group (^Δ^ *p* < 0.05, ^ΔΔ^ *p* < 0.01). DSS + STD − DIC mice fed standard laboratory diet (STD), DSS + MC1 − DIC mice fed STD and *Llb. fermentum* MC1, DSS + EPS − DIC mice fed STD and EPSs of *Llb. fermentum* MC1.

**Table 3:** Differential blood count of dextran sulfate sodium (DSS)-induced colitis (DIC) mice, treated and non-treated with the strain *Limosilactobacillus fermentum* MC1 or its EPSs.

Groups	Neutrophils (%)	Lymphocytes (%)	Monocytes (%)	Eosinophils (%)	Basophils (%)
DSS + STD	19.98 ± 5.66	69.43 ± 3.07	16.46 ± 2.72	0.28 ± 0.28	0.00 ± 0.00
DSS + MC1	2.60 ± 0.26 **	62.77 ± 2.61	34.37 ± 2.76 **	0.27 ± 0.27	0.00 ± 0.00
DSS + EPS	1.37 ± 0.74 **	76.80 ± 3.50 ^Δ^	20.37 ± 2.86 ^Δ^	1.47 ± 1.47	0.00 ± 0.00

Statistically different compared to DSS + STD group (** *p* < 0.01). Statistically different compared to DSS + MC1 group (^Δ^ *p* < 0.05). DSS + STD: DIC mice fed standard laboratory diet (STD); DSS + MC1: DIC mice fed STD and *Llb. fermentum* MC1; DSS + EPS: DIC mice fed STD and EPSs of *Llb. fermentum* MC1.

## Data Availability

The original contributions presented in this study are included in the article/Supplementary Materials. Further inquiries can be directed to the corresponding author.

## Supplementary Materials

The following supporting information can be downloaded at https://www.mdpi.com/article/10.3390/ijms27052321/s1.
