# Supplementary material for: In Vivo Evaluation of the Effect of Limosilactobacillus fermentum MC1 and Its EPSs on the Microbiota and Inflammatory Processes in the Mouse Intestine
Source: Int J Mol Sci. 2026 Mar 1;27(5):2321. doi: 10.3390/ijms27052321 (PMC12986256; doi:10.3390/ijms27052321)
Supplement: Supplementary file 1 [file ijms-27-02321-s001.zip › ijms-4146796-supplementary.pdf]

SUPPLEMENTARY DATA

In vivo evaluation of the effect of *Limosilactobacillus fermentum* MC1 and its EPSs on the microbiota and inflammatory processes in the mouse intestine

Nina Čuljak[a]*, Nada Oršolić*[b]*, Dyana Odeh*[b]*, Andreja Leboš Pavunc*[a]*, Katarina Butorac*[a]*, Martina Banić*[a]*, Jasna Novak*[a*]*, Kate Šešelja*[c]*, Mirela Baus Lončar*[c]*, Snježana Ramić*[d]*, Tanja Jurkin*[e]*, Jagoda Šušković*[a] *& Blaženka Kos*[a]

[a]Department of Biochemical Engineering, University of Zagreb Faculty of Food Technology and Biotechnology, Pierottijeva 6, 10 000 Zagreb, Croatia

[b]Department of Animal Physiology, University of Zagreb Faculty of Science, Horvatovac 102a, 10 000 Zagreb, Croatia

[c]Department of Molecular Medicine, Ruđer Bošković Institute, Bijenička 54, 10 000 Zagreb, Croatia

[d]Department of Oncological Pathology, University Hospital for Tumors, Sestre Milosrdnice University Hospital Centre, Ilica 197, 10000 Zagreb, Croatia

[e]Radiation Chemistry and Dosimetry Laboratory, Ruđer Bošković Institute, Bijenička 54, 10 000 Zagreb, Croatia

*Corresponding author: jasna.novak@pbf.unizg.hr

Table S1. Monitored mouse body weight changes during the experiments in control, healthy and in dextran sulfate sodium (DSS)-induced colitis (DIC) model groups

Group	DSS			HEALTHY		
	Initial weight (g)	Final weight (g)	Weight difference	Initial weight (g)	Final weight (g)	Weight difference
**STD**	28.47 ± 0.30	22.56 ± 2.68	-7.16 ± 2.03	30.13 ± 0.73	28.90 ± 1.17	-1.23 ± 0.49****
**MC1**	24.92 ± 0.49	19.77 ± 2.44	-4.62 ± 2.15	24.90 ± 1.26	24.83 ± 1.86	-0.07 ± 0.94**
**EPS**	26.97 ± 0.75	21.42 ± 2.69	-4.71 ± 1.93	27.05 ± 0.38	25.20 ± 0.33	-1.85 ± 0.05**

*Statistically different in weight compared to the DSS group (** $p < 0.01$, **** $p < 0.0001$)*

STD – mice fed standard laboratory diet (STD), MC1 – mice fed STD and Limosilactobacillus fermentum MC1, EPS – mice fed STD and EPSs of strain Llb. fermentum MC1

Table S2. Hematological parameters in healthy mice

Hematological parameters	STD	MC1	EPS
**Erythrocytes (10^{12}/L)**	6.94 ± 0.85	7.51 ± 0,38	8.27 ± 0.79
**Leukocytes (10^9/L)**	4.78 ± 0.15	6.76 ± 0.48	5.95 ± 0.71
**Hemoglobin (g/L)**	105.00 ± 12.58	112.00 ± 6.76	124.83 ± 12.54
**Hematocrit (%)**	0.43 ± 0.04	0.46 ± 0.02	0.50 ± 0.04
**Erythrocyte volume (fL)**	62.70 ± 1.27	61.63 ± 0.38	60.20 ± 0.50
**Mean corpuscular hemoglobin (pg)**	15.13 ± 0.05	14.87 ± 0.10	15.07 ± 0.05
**Mean corpuscular hemoglobin concentration (g/L)**	235.67 ± 4.84	241.33 ± 2.48	250.33 ± 2.39
**Erythrocyte distribution (%)**	14.00 ± 0.19	13.60 ± 0.12	14.53 ± 0.38
**Thrombocytes (10^9/L)**	301.00 ± 71.83	444.50 ± 32.63	604.33 ± 57.58**
**Thrombocyte volume (fL)**	6.90 ± 0.24	6.73 ± 0.05	6.80 ± 0.09

*Statistically different compared to the STD group (** $p < 0.01$)*

STD – healthy mice fed standard laboratory diet (STD), MC1 – healthy mice fed STD and Limosilactobacillus fermentum MC1, EPS – healthy mice fed STD and EPSs of strain Llb. fermentum MC1

Table S3. Differential blood count of healthy C57BL/6 mice not treated or treated with strain *Limosilactobacillus fermentum* MC1 or exopolysaccharides (EPS)

Group	Neutrophils (%)	Lymphocytes (%)	Monocytes (%)	Eosinophils (%)	Basophils (%)
**STD**	11.73 ± 0.68	84.60 ± 0.91	2.07 ± 1.48	1.60 ± 1.24	0.00 ± 0.00
**MC1**	10.03 ± 1.93	88.27 ± 2.44	0.93 ± 0.36	0.77 ± 1.19	0.00 ± 0.00
**EPS**	10.43 ± 3.04	79.70 ± 2.87	9.87 ± 4.56	0.00 ± 0.00*	0.00 ± 0.00

Statistically different compared to the STD group ($p < 0.05$)*

STD – healthy mice fed standard laboratory diet (STD), MC1 – healthy mice fed STD and Llb. fermentum MC1, EPS – healthy mice fed STD and EPSs of strain Llb. fermentum MC1

Table S4. Primers used for qPCR analysis with optimized conditions

Target gene	Primer sequence (5′-3′)	qPCR conditions*
GRP94	AAGAATGAAGGAAAAACAGGACA<br>AAACAAATGGAGAAGATTCCGCC	58 °C, 3 mM
IGF1R	CCAAGTTACCAGAAGGGCTAAT<br>GGTAGGGACAATACAGGCTAAAT	59 °C, 3 mM
IL1α	CCTTACACCTACCAGAGTGA<br>AACCAAGTGGTGCTGAGATA	65 °C, 3 mM
IL1β	ATGGGCAACCACTTACCTATTT<br>GTTCTAGAGAGTGCTGCCTAATG	64 °C, 3 mM
IL6	GATAAGCTGGAGTCACAGAAGG<br>TTGCCGAGTAGATCTCAAAGTG	59 °C, 3.5 mM
MCP1	CCTGGATCGGAACCAAATGA<br>CGGGTCAACTTCACATTCAAAG	62 °C, 3 mM
NOX2	ACTCCTTGGGTCAGCACTGG<br>GTTCCTGTCCAGTTGTCTTCG	62 °C, 3 mM
TLR4	TGGTCAGTGTGATTGTGGTATC<br>GCTTTCTCCTCTGCTGTACTT	58 °C, 3 mM
TNFα	GTCTCAGAATGAGGCTGGATAAG<br>CATTGCACCTCAGGGAAGAA	63 °C, 2.5 mM
TGFβ	CTTTAGGAAGGACCTGGGTTG<br>GTGTGTCCAGGCTCCAAATA	66 °C, 3 mM
CD68	CTCTTGCTGCCTCTCATCATT<br>CTGGTAGGTTGATTGTCGTCTG	58 °C, 2.5 mM
CHOP	TTGAGCCTAACACGTCGATTAT<br>CACTTCCTTCTGGAACACTCTC	58 °C, 3 mM
BCL2	TCCAGCCACCTTCTTTCTATG<br>GTTTCACCATGTTGCCTCTTAC	61 °C, 3 mM
AIFM1	CGGGAGGTCAAGTCAATTACAG<br>GGAACAGCTGGATCACTTCTATG	61 °C, 3.5 mM

**primer annealing temperature and $MgCl_2$ concentration*

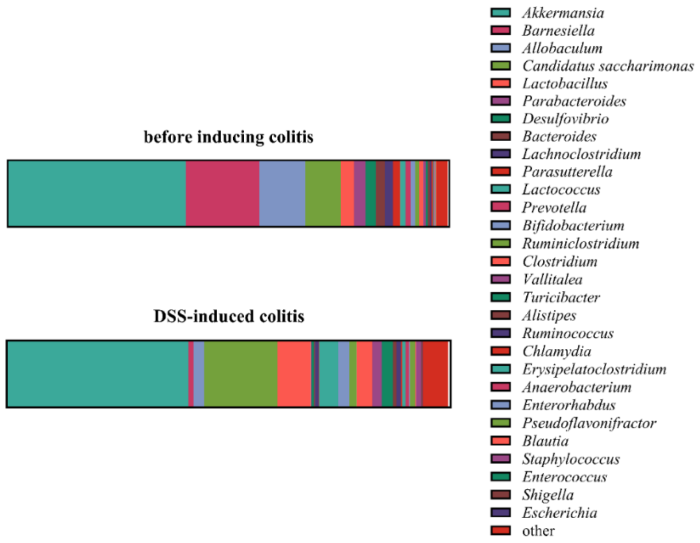

Figure S1. Relative abundance (%) of bacteria at genus level in mice feces before and after dextran sulfate sodium (DSS)-induced colitis (DIC)

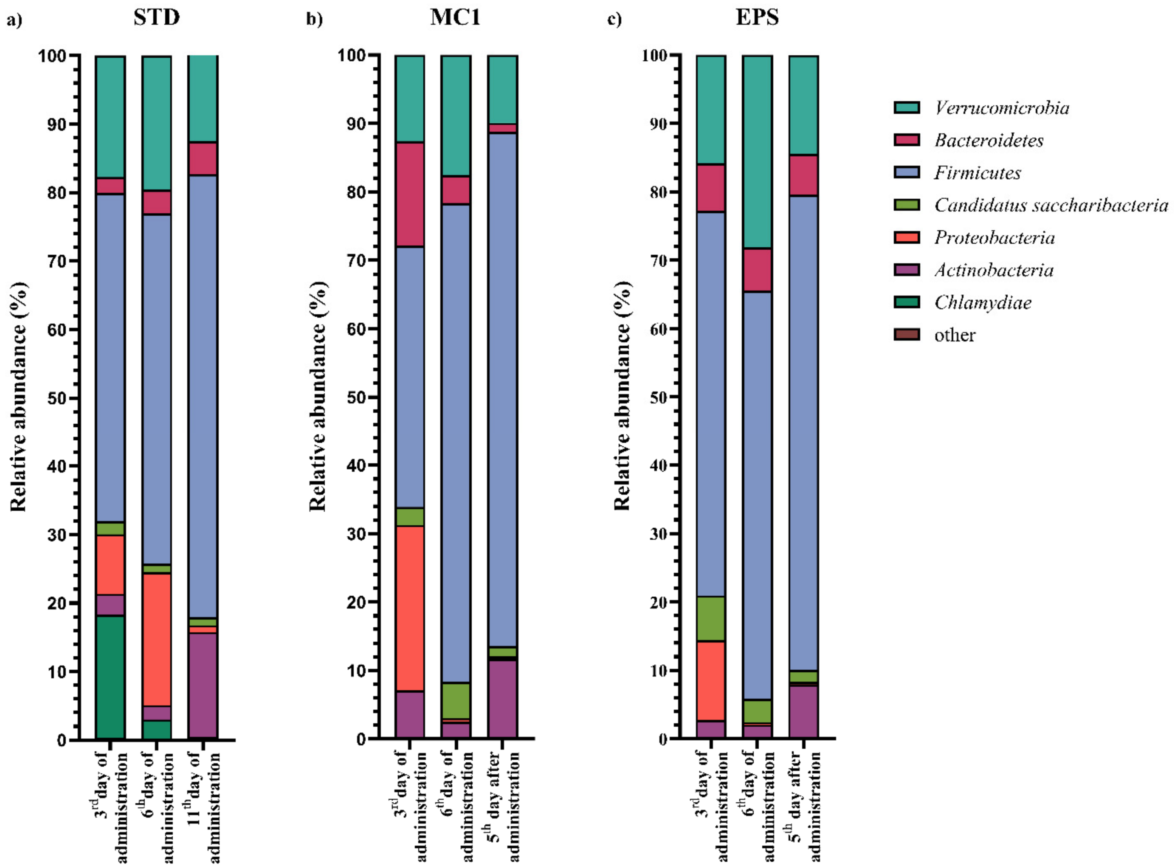

Figure S2. Relative abundance (%) of bacteria at the phylum level in intestinal microbiota of mice treated with dextran sulfate sodium (DSS)-induced colitis (DIC) a) on the 3rd, 6th, and 11th day of feeding with standard laboratory diet (control); on the 3rd and 6th day of feeding and on the 5th day after termination of feeding with b) strain *Llb. fermentum* MC1 and c) EPSs of *Llb. fermentum* MC1

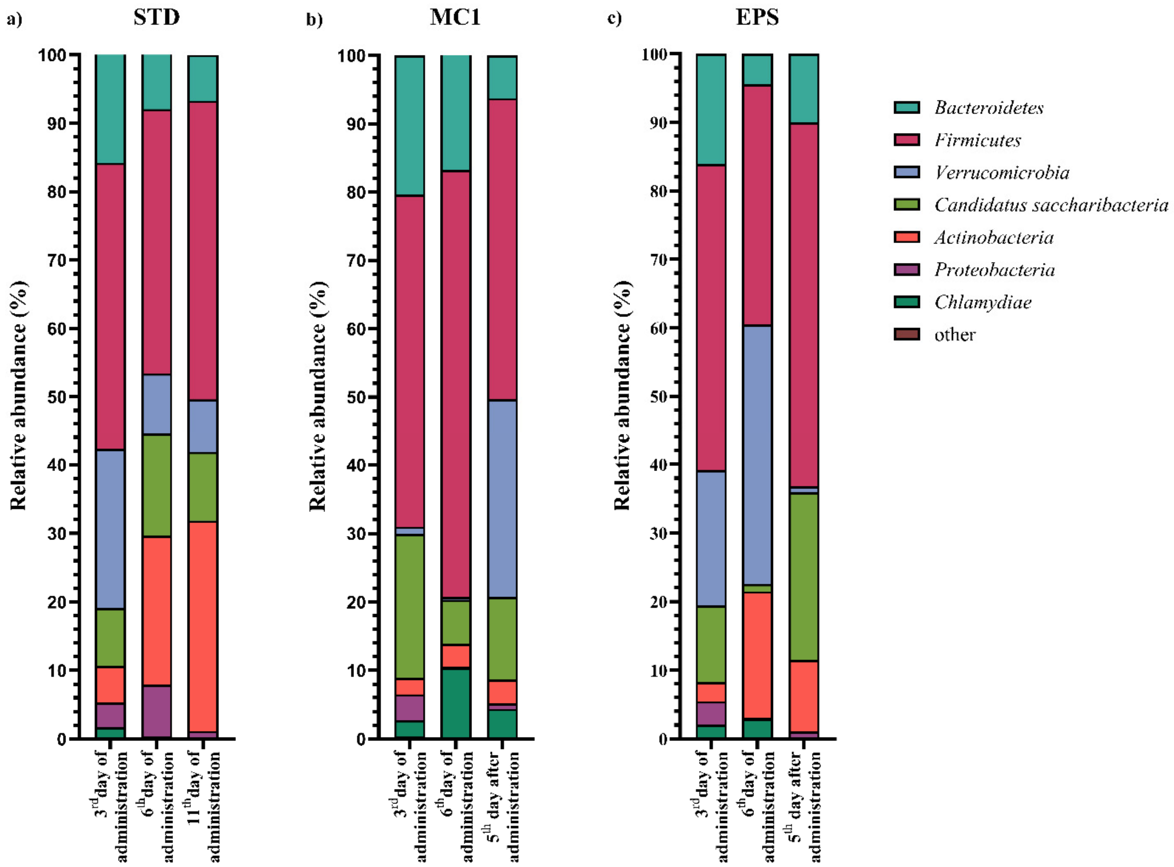

Figure S3. Relative abundance (%) of bacteria at the phylum level in intestinal microbiota of healthy mice a) on the 3rd, 6th, and 11th day of feeding with standard laboratory diet (control); on the 3rd and 6th day of feeding and on the 5th day after termination of feeding with b) strain *Llb. fermentum* MC1 and c) EPSs of *Llb. fermentum* MC1

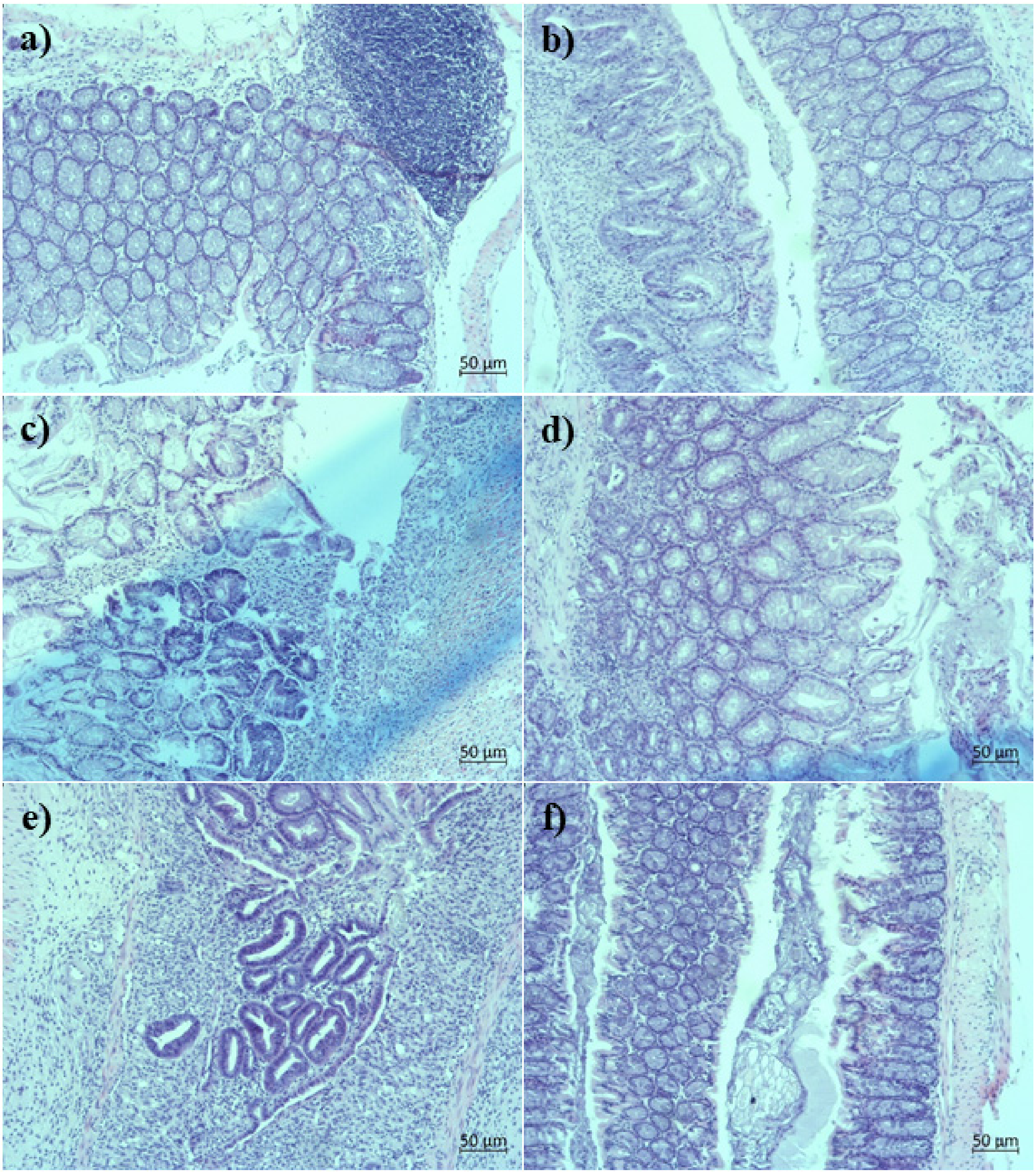

Figure S4. Effect of DSS and probiotic strain *Limosilactobacillus fermentum* MC1 and its EPSs on pathohistological changes in the colonic mucosa

*Representative images are shown: **a)** normal colonic mucosa with lymphocyte infiltrate, **b)** control group treated with DSS: dysplasia visible on the left; on the right is healthy intestinal mucosa, **c)** control group treated with DSS: visible carcinoma in situ, **d)** group treated with EPSs: on the right is mild dysplasia with superficial ulceration while on the left is normal intestinal mucosa, **e)** group treated with EPSs: visible carcinoma in situ, **f)** group treated with Llb. fermentum MC1: on the left is healthy intestinal mucosa while on the right ischemic changes are visible.*

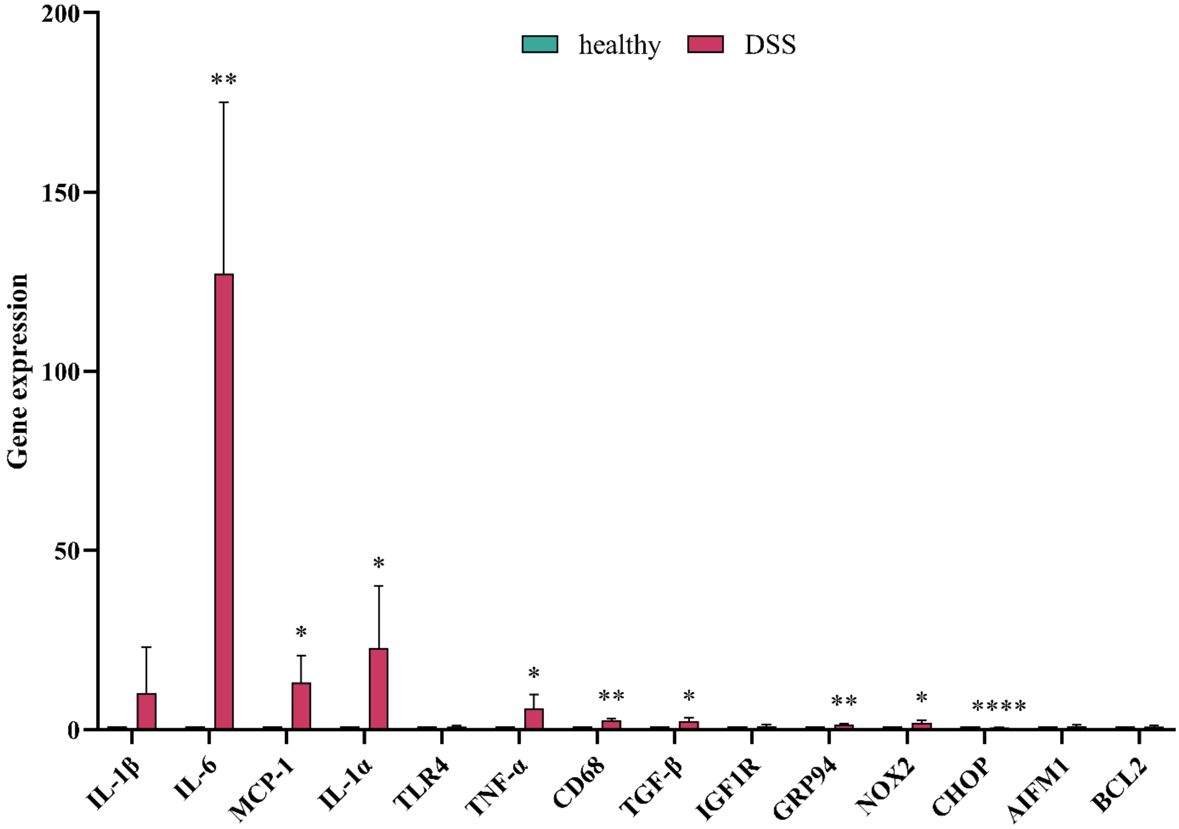

Figure S5. Expression of genes related to inflammation (IL-1β, IL-6, MCP-1, IL-1α, TLR4, TNF-α, CD68, TGF-β), apoptosis (BCL2, AIFM1, IGF1R), endoplasmic (CHOP, GRP94) and oxidative stress (NOX2) in the colon of mice upon colitis induction. Expression of monitored genes was compared relative to expression in healthy mice (level set to 1).

Statistically different compared to the healthy group ($p < 0.05$, ** $p < 0.01$, **** $p < 0.0001$)*

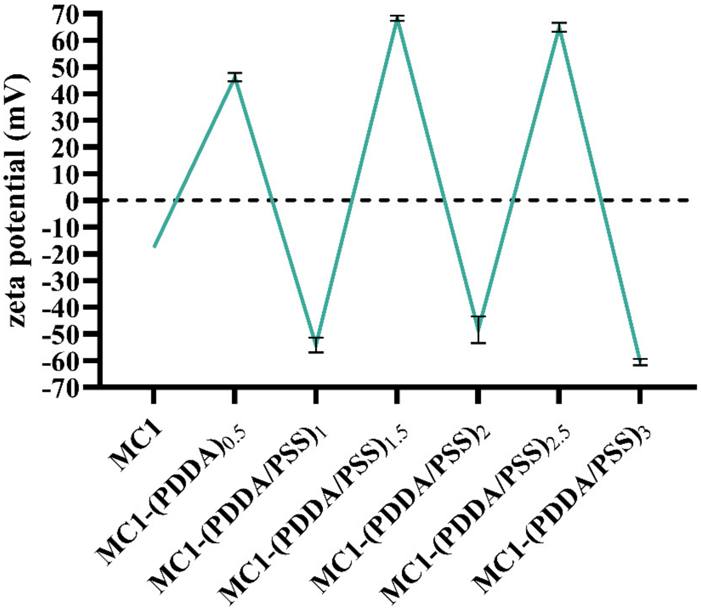

Figure S6. Zeta potential during the encapsulation of *Limosilactobacillus fermentum* MC1 with PDDA/PSS with three layers

PDDA – poly(diallyldimethylammonium chloride)

PSS – poly(sodium styrenesulfonate)
